# Prevent2Protect Project: Regulatory Focus Differences in Sexual Health Knowledge and Practices

**DOI:** 10.1007/s10508-023-02536-3

**Published:** 2023-01-26

**Authors:** David L. Rodrigues, Richard O. de Visser, Diniz Lopes, Marília Prada, Margarida V. Garrido, Rhonda N. Balzarini

**Affiliations:** 1 Iscte-Instituto Universitário de Lisboa, CIS-Iscte, Av. das Forças Armadas, 1649-026 Lisbon, Portugal; 2 Brighton and Sussex Medical School, Brighton, UK; 3 Department of Psychology, Texas State University, San Marcos, TX USA; 4 The Kinsey Institute, Indiana University, Bloomington, IN USA

**Keywords:** Regulatory focus, Prevention, Promotion, Sexuality, STI, Sexual health

## Abstract

The way people perceive risks and make decisions about their health is regulated by two motivational systems—prevention of harm or promotion of pleasure. People more focused on prevention strive to avoid negative outcomes and enact more health-protective behaviors. In contrast, people more focused on promotion strive to attain positive outcomes and take more risks with their health. Building upon recent evidence extending this framework to sexual behaviors and health, we conducted a pre-registered online survey (OSF) with Portuguese and Spanish adults (*N* = 742; *M*_age_ = 31.42, *SD* = 9.16) to examine whether self-reported STI knowledge and sexual health practices differ based on predominant regulatory focus. This study was part of the Prevent2Protect project (OSF). Results showed that prevention-focused participants had heard about more STIs and retrieved more of their knowledge from scientific sources but had been tested for fewer STIs in the past. In contrast, promotion-focused participants indicated they had specific knowledge about more STIs, retrieved more of their knowledge from medical and peer sources, and had been tested for more STIs in the past. They also reported more frequent STI testing, using free testing facilities or asking their family practice doctor to get tested, more routine sexual health check-ups, and more use of other contraceptive methods, such as birth control pills. These results remained unchanged after controlling for demographic differences. Overall, our findings showed that different motives in sexuality shape sexual health knowledge and sexual health practices.

**Supplementary Information:**

The online version contains supplementary material available at 10.1007/s10508-023-02536-3.

## Introduction

Sexually transmitted infections (STIs) remain a significant public health concern, with a resurgence of reported cases in recent years (for reviews, see Scott-Sheldon & Chan, 2020; Sukhija-Cohen et al., 2019). Health communication, behavioral interventions, and sexual education programs have been shown to improve sexual health outcomes (for reviews, see Friedman et al., 2016; Soe et al., 2018; Whiting et al., 2019; Zhang et al., 2021). For example, intervention programs in Portugal (e.g., Carvalho et al., 2016; Costa et al., 2017) and Spain (e.g., Ballester-Arnal et al., 2017; Espada et al., 2015) have been shown to improve knowledge about STIs, sexual health attitudes and self-efficacy, risk perception, and condom use. Despite these efforts, some people still lack adequate knowledge and sometimes decide to forgo sexual health behaviors on a regular basis, therefore increasing the risk of STI acquisition. Indeed, research has shown that people in Portugal and Spain have an overall low knowledge about STIs, many report condomless sex practices, and most have never been tested for STIs (e.g., Espada et al., 2015, 2016; Rodrigues et al., 2020; Santos et al., 2018). Furthermore, research shows that in both countries in recent years, condom use intentions have been decreasing, alongside an increased rate of condomless sex (Alvarez-Bruned et al., 2015; Ballester-Arnal et al., 2022; Giménez-García et al., 2022; Reis et al., 2018).

Diverse theoretical models aim to predict sexual health behaviors and reduce STI rates (for a review, see Glanz et al., 2015). For example, people are more likely to use condoms when they enact preparatory behaviors (e.g., buy condoms), feel more susceptible to STIs, or have more confidence in the correct use of condoms (Bryan et al., 2006; Carvalho et al., 2015; Espada et al., 2016; Martin-Smith et al., 2018; Reid & Aiken, 2011). Research has also discussed the importance of self-control to regulate perceptions and behaviors when making sexual health decisions (e.g., Rodrigues et al., 2019a, 2019b, 2019c; Wiederman, 2004). From a regulatory focus perspective (for a review, see Higgins, 2015), this regulation operates through two distinct motivational systems—prevention focus and promotion focus—that shape how people perceive the context and pursue their goals. People more focused on prevention are driven by safety, are more aware of threats, aim to avoid adverse outcomes, and perceive themselves to have more control over their behaviors, whereas people who are more focused on promotion are driven by pleasure, seek to attain positive outcomes, take risks, and believe they have more control over the outcomes of their behaviors (e.g., Guo & Spina, 2015; Higgins et al., 2001; Langens, 2007; Lemarié et al., 2019). Research has shown that people who are more focused on prevention (vs. promotion) are more aware of health threats, less likely to take risks with their health, and more likely to enact protective health behaviors (e.g., Ferrer et al., 2017; Fuglestad et al., 2013; Zou & Scholer, 2016). These individual motives also influence the type of sources sought for health information in threatening contexts such as the COVID-19 pandemic: people who were more focused on prevention were also more likely to have consulted scientific sources (e.g., scientific reports), whereas people who were more focused on promotion retrieved information from peers and the media (Rodrigues, 2022).

A similar pattern has been recently observed in the sexuality domain. Aligned with other studies in the health domain, both Rodrigues and colleagues (Rodrigues, 2022; Rodrigues & Lopes, 2022; Rodrigues et al., 2020, 2022, 2019a, 2019b, 2019c), and Evans-Paulson et al., (2022), found that people who are more focused on prevention tend to perceive more threats to their sexual health. These people also have more positive attitudes toward condoms, stronger intentions to use condoms, are more likely to have used condoms with casual partners, and have more control over condom use behavior (i.e., a greater focus on the prevention of harm). In contrast, people who are more focused on promotion report using condoms less frequently, having stronger intentions to take health risks and get tested for STIs, and being more sexually satisfied (i.e., a greater focus on the promotion of pleasure). However, research supporting the importance of regulatory focus on broader sexual health knowledge (e.g., STI knowledge, sources of information) and practices (e.g., STI testing, contraceptive use) is still limited, and findings are yet to be generalized to different cultural contexts.

### Overview of the Study

As part of the Prevent2Protect project (for details, see OSF), we conducted a cross-sectional study with people living in Portugal and Spain to explore *if* and *how* differences in regulatory focus shape self-reported knowledge about STIs and sexual health practices and routines. All hypotheses, materials, and procedures were pre-registered (see OSF).

Building upon the available evidence (Evans-Paulson et al., 2022; Rodrigues, 2022; Rodrigues et al., 2020, 2022, 2019a, 2019b, 2019c), we expected prevention-focused (vs. promotion-focused) participants to indicate knowing a higher percentage of STIs (H1), having retrieved more of their knowledge from scientific or medical (vs. peer) sources (H2), and recommend a higher frequency of testing across different STIs (H3). In contrast, we expected promotion-focused (vs. prevention-focused) people to indicate having been tested for a higher percentage of STIs (H4) and having been diagnosed with more of those STIs (H5).

We also conducted a series of exploratory analyses to examine if regulatory focus was associated with past sexual health practices. Specifically, we explored if participants differed in how frequently they got tested for STIs in the past, where they chose to get tested, how often they attended routine sexual health check-ups, and if they used contraceptive methods other than condoms. Lastly, we explored differences according to demographic variables and examined if our results were consistent after controlling for those variables to determine the generalizability of our findings. Particular attention was given to gender, given that women (vs. men) tend to have more sexual health knowledge (e.g., Weinstein et al., 2008) but report less condom use self-efficacy and often feel they have less control over their sexual encounters (e.g., Farmer & Meston, 2006); sexual identity, given that LGBTQI + men tend to use condoms more frequently than women and heterosexual men (e.g., Fetner et al., 2020); education, given that less educated people are more likely to have condomless sex (Rodrigues et al., 2020); and country, given that Spanish people report more frequent condom use, whereas Portuguese people report more self-efficacy in negotiating condom use and using condoms in risky situations (e.g., Muñoz-Silva et al., 2007).

## Method

### Participants and Procedure

The Prevent2Protect project was approved by the Ethics Committee at Iscte-Instituto Universitário de Lisboa (#70/2021). A power analysis using G*Power (Faul et al., 2009), considering a medium effect size (*f* = 0.10) and 95% power, indicated that at least 624 participants would be needed for this study. We increased this estimation by 30% to account for incomplete surveys and participants not meeting the inclusion criteria, resulting in a target sample of 812 participants.

The survey was originally developed in English and then translated to Portuguese and Spanish. When measures were unavailable in these languages, we used the back-translation process (Colina et al., 2017). Data were collected during October and November 2021 on a survey hosted in Qualtrics. Participants were recruited on the Clickworker online platform and informed that completed surveys would be compensated with 5€ on their user account. To be eligible, participants had to be at least 18 years old, have already engaged in sexual activity, be currently single (defined as not having a romantic relationship with a significant other), and live in Portugal and speak Portuguese or live in Spain and speak Spanish. We restricted our analyses to people who were single because these people are more likely to have sex with concurrent partners and may be more exposed to sexual health risks. These pre-screen questions were included at the beginning of the survey, and participants who failed to meet the eligibility requirements were redirected to the end of the survey and thanked for their time. Apart from these pre-screen measures, all other responses were nonmandatory due to their sensitive nature. Participants were reminded if they left any unanswered questions but were allowed to proceed with the survey.

Of the 925 eligible participants, only completed surveys from 812 participants who met the inclusion criteria were retained for the analyses. As commonly employed in the literature (e.g., Berinsky et al., 2014; Curran, 2016), this study included attention check items. Two items served as attention checks and asked participants to select a particular answer choice for that question (“Please select the option “3”. This is not a trick question.”). We excluded 29 participants who failed at least one of the attention checks. Additionally, we asked participants how much attention they paid while completing the survey (1 = *No attention* to 4 = *Very close attention*) and if they wanted their responses to be analyzed (1 = *I want to keep my responses for analyses*, 2 = *I want to withdraw my responses from this study*). Nine participants were removed because they reported little or no attention, and four participants asked to withdraw their responses. Lastly, we removed 28 participants because they had no predominant regulatory focus in sexuality (see below) and were too few to comprise a reliable comparison group.

The final sample included 742 participants. Overall demographic characteristics and differences between regulatory focus groups are detailed in Table 1. Participants were on average 31 years old, and most identified as White, women, heterosexual, from Spain, resided in metropolitan areas, had a university degree, were workers, and were coping on their present incomes. Comparisons between regulatory focus groups showed differences in gender, *p* = .016, sexual identity, *p* = .023, and socioeconomic status, *p* = .034. We found a higher proportion of promotion-focused participants who were women or were bisexual, and a higher proportion of prevention-focused participants who were men or were heterosexual. Results also showed a higher proportion of prevention-focused participants who preferred not to indicate their socioeconomic status.

**Table 1:** Demographic characteristics

	Overall (*N* = 742)	Regulatory focus group		
		Promotion focus (*n* = 378)	Prevention focus (*n* = 364)	Comparisons
	*M* (*SD*) or *n* (%)	*M* (*SD*) or *n* (%)	*M* (*SD*) or *n* (%)	*t* (*d*) or χ^2^ (*V*)
Age (min = 18, max = 62)	31.42 (9.16)	31.94 (8.67)	30.88 (9.63)	1.58 (0.12)
Ethnic background				7.76 (0.10)
Arab	6 (0.8)	4 (1.1)	2 (0.5)	
Asian	2 (0.3)	2 (0.5)	0 (0.0)	
Black	28 (3.8)	16 (4.2)	12 (3.3)	
Latinx	118 (15.9)	69 (18.3)	49 (13.5)	
Mixed race	6 (0.8)	4 (1.1)	2 (0.5)	
White	579 (78.0)	282 (74.6)	297 (81.6)	
Prefer not to answer	3 (0.4)	1 (0.3)	2 (0.5)	
Gender				10.39^*^ (0.12)
Man	316 (42.6)	143^b^ (37.8)	173^a^ (47.5)	
Non-binary	7 (0.9)	2^a^ (0.5)	5^a^ (1.4)	
Woman	418 (56.3)	233^a^ (61.6)	185^b^ (50.8)	
Prefer not to answer	1 (0.1)	0 (0.0)	1 (0.3)	
Sexual identity				13.06^*^ (0.13)
Asexual	3 (0.4)	0^a^ (0.0)	3^a^ (0.8)	
Bisexual	114 (15.4)	73^a^ (19.3)	41^b^ (11.3)	
Heterosexual	578 (77.9)	279^b^ (73.8)	299^a^ (82.1)	
Lesbian/Gay	39 (5.3)	22^a^ (5.8)	17^a^ (4.7)	
Pansexual	6 (0.8)	3^a^ (0.8)	3^a^ (0.8)	
Queer	2 (0.3)	1^a^ (0.3)	1^a^ (0.3)	
Country				1.10 (0.04)
Portugal	328 (44.2)	160 (42.3)	168 (46.2)	
Spain	414 (55.8)	218 (57.7)	196 (53.8)	
Residence				5.04 (0.08)
Metropolitan area	461 (62.1)	248 (65.6)	213 (58.5)	
Rural area	90 (12.1)	40 (10.6)	50 (13.7)	
Small town	4 (0.5)	2 (0.5)	2 (0.5)	
Suburban area	186 (25.1)	88 (23.3)	98 (26.9)	
Prefer not to answer	1 (0.1)	0 (0.0)	1 (0.3)	
Completed education				6.67 (1.00)
Primary or secondary school	14 (1.9)	6 (1.6)	8 (2.2)	
High school	221 (29.8)	102 (27.0)	119 (32.7)	
Professional training	7 (0.9)	3 (0.8)	4 (1.1)	
University degree	330 (44.5)	181 (47.9)	149 (40.9)	
Post-graduate (Master’s; Ph.D.)	168 (22.6)	86 (22.8)	82 (22.5)	
Prefer not to answer	2 (0.3)	0 (0.0)	2 (0.5)	
Occupation				7.81 (0.10)
Retired	5 (0.7)	4 (1.1)	1 (0.3)	
Stay-at-home parent	7 (0.9)	4 (1.1)	3 (0.8)	
Student (part or full time)	213 (28.7)	96 (25.4)	117 (32.1)	
Unemployed	82 (11.1)	39 (10.3)	43 (11.8)	
Working (part or full time)	429 (57.8)	233 (61.6)	196 (53.8)	
Prefer not to answer	6 (0.8)	2 (0.5)	4 (1.1)	
Socioeconomic status				12.03^*^ (0.13)
Very difficult on present income	52 (7.0)	26^a^ (6.9)	26^a^ (6.9)	
Difficult on present income	162 (21.8)	90^a^ (23.8)	72^a^ (19.8)	
Coping on present income	337 (45.4)	161^a^ (42.6)	176^a^ (48.4)	
Comfortable on present income	154 (20.8)	86^a^ (22.8)	68^a^ (18.7)	
Very comfortable on present income	26 (3.5)	14^a^ (3.7)	12^a^ (3.3)	
Prefer not to answer	11 (1.5)	1^b^ (0.3)	10^a^ (2.7)	

Different superscripts for regulatory focus groups indicate significant differences in column proportions with Bonferroni correction at *p* < .050. ^***^p ≤ .001, ^**^p ≤ .010, ^*^p ≤ .050

## Measures

### Regulatory Focus in Sexuality

Regulatory focus was assessed using a measure developed by Rodrigues et al., (2019) to assess motives for prevention (three items; e.g., “Not being careful enough in my sex life has gotten me into trouble at times” [reverse-coded]) and promotion (six items; e.g., “I am typically striving to fulfill my desires with my sex life”) in sexuality. Responses were given on 7-point rating scales (1 = *Not at all true of me* to 7 = *Very true of me*). Items in each subscale were mean aggregated, with higher scores indicating a greater focus on prevention (*α* = .70) or promotion (*α*  = .82). Aligned with previous cross-national studies (e.g., Evans-Paulson et al., 2022; Rodrigues, 2022), a confirmatory factor analysis using Mplus 7 (Muthén & Muthén, 2012) with robust maximum likelihood estimation (Yuan & Bentler, 2000) showed good fit indexes in both samples, χ^2^(25) ≥ 40.87, CFI ≥ .95, TLI ≥ .93, SRMR ≤ .05, RMSEA ≤ .07 (Byrne, 2012), with moderate to high standardized regression paths for both prevention, .44 ≥  λ ≥ .83, and promotion, .48 ≥ λ ≥ .82, subscales.

To compare participants based on their predominant regulatory focus in sexuality, we first computed an index by subtracting promotion from prevention scores (Rodrigues et al., 2019b). Positive scores indicated a predominant focus on prevention in sexuality (*n* = 378), negative scores indicated a predominant focus on promotion in sexuality (*n* = 364), and scores equal to zero indicated that participants had no predominant regulatory focus in sexuality (*n* = 28). Given the small number of participants in this latter group, we were unable to conduct reliable comparisons and therefore removed them from the final sample.

### Self-Reported STI Knowledge and Sources of Information

Participants were shown a list of 13 STIs (e.g., HIV, chlamydia, syphilis, trichomoniasis). For each, they were asked if they had heard about or had specific knowledge about the STI (1 = *I’ve never heard about this STI*; 2 = *I’ve heard about this STI, but I have no knowledge about it*; 3 = *I have specific knowledge about this STI*). We computed the percentages for each response option, averaged across STIs.

When participants indicated having specific knowledge, we additionally asked them where they got their information. We provided a list of 13 possible sources for each STI, and participants were allowed to select multiple options. Sources were then grouped into self and peer sources (including same-sex friends, different-sex friends, parents, romantic partners, casual sex partners, social media, and other Internet sources [e.g., google search, general websites]), medical sources (including STI screening tests, family practice doctor, family planning consultations, other specialists [e.g., documentaries, interviews with specialists], and the National Department of Health website), and scientific sources (including sexual education materials, scientific papers, scientific talks). We computed the percentage of each source category and averaged it across STIs.

### Past STI Testing and Diagnosis

Participants were also asked if they have been tested for (1 = *No*; 2 = *Yes*; 3 = *Don’t know*), or diagnosed with (1 = *No*; 2 = *Yes*), each STI. In both cases, we computed the percentages for each response option, averaged across STIs. For the second variable, we also computed the proportion of participants diagnosed with at least one STI.

### Recommended Frequency of STI Testing

We asked participants how frequently they think people should get tested for each STI (1 = *Never* to 7 = *After each new casual partner*). We computed an overall mean across STIs, with higher scores indicating the belief that people should be tested more frequently.

### Sexual Health Practices

Participants were asked how often they get tested for STIs (1 = *I was never tested before* to 7 = *I get tested frequently*) and how often they have routine sexual health check-ups (1 = *I never went* to 4 = *More than once a year*). We also asked participants where they typically get tested and provided the options: “never tested,” “free STI testing facilities,” “asked my family practice doctor,” “family planning consultations,” “self-testing kit,” and “other (please specify).” Lastly, we asked participants if they currently use contraceptive method(s) other than condoms and provided four options: “no other method,” “birth control pill,” “intrauterine device (IUD),” and “other (please specify).” In both cases, participants were allowed to select multiple options, and we computed the proportion of participants who selected each testing location and contraceptive method (1 = *No*; 2 = *Yes*).

### Data Analytic Plan

We computed four separate mixed ANOVAs to examine differences in STI knowledge, sources of information, past testing, and past diagnosis (within-participants factors) according to regulatory focus in sexuality (between-participants factor). When differences were found, we computed post-hoc comparisons with Bonferroni adjustment. We also examined group differences in the recommended frequency of STI testing using a *t*-test and group differences in the proportion of sources of information used using Wald tests.

Additionally, we used *t*-tests to explore if both groups differed in the frequency of past STI testing and sexual health check-ups and Wald tests to determine if they differed in testing location and contraceptive method use. Lastly, we used *t*-tests, and Wald tests to examine differences according to gender (men vs. women), sexual identity (heterosexual vs. LGBTQI +), education (≤ 12 years vs. > 12 years), and country (Portugal vs. Spain). When differences were found, we re-run our analyses entering variables as covariates.

## Results

### Preliminary Analyses

Overall estimated marginal means are presented in Table 2. On average, participants had only heard about half of the STIs, retrieved more of their STI knowledge from scientific sources, had never been tested for most STIs, and were rarely diagnosed with any of the STIs. Participants also considered that people should get tested for STIs somewhat frequently. Details for each STI separately are presented as Supplementary Materials and shared on the Prevent2Protect’s OSF page.

**Table 2:** Results for the main variables across STIs

	Overall		Regulatory focus group		
			Promotion focus	Prevention focus	Comparisons
	% (*SE*) or *M* (*SE*)	Range	% (*SE*) or *M* (*SE*)	% (*SE*) or *M* (*SE*)	
Self-reported STI knowledge					
Never heard about any	27.96^b^ (0.65)	[0.00; 100.00]	27.64 (0.91)	28.28 (0.93)	*ns*
Only heard about any	50.11^a^ (0.92)	[0.00; 100.00]	47.62 (1.29)	52.60 (1.31)	^**^
Have specific knowledge about any	21.78^c^ (0.92)	[0.00; 100.00]	24.70 (1.29)	19.04 (1.31)	^**^
Sources of information					
Self and peer sources	4.23^a^ (0.25)	[0.00; 42.86]	5.01 (0.34)	3.46 (0.36)	^**^
Medical sources	4.98^a^ (0.30)	[0.00; 41.54]	5.73 (0.41)	4.22 (0.43)	^*^
Scientific sources	5.18^a^ (0.64)	[0.00; 100.00]	3.82 (0.88)	6.55 (0.93)	^*^
Past STI testing					
Never tested for any	69.38^a^ (1.45)	[0.00; 100.00]	65.02 (2.03)	73.73 (2.07)	^**^
Tested for any	18.22^b^ (1.01)	[0.00; 100.00]	22.51 (1.42)	13.93 (1.44)	^***^
Unsure of testing for any	10.04^c^ (0.85)	[0.00; 100.00]	10.30 (1.19)	9.78 (1.21)	*ns*
Past STI diagnosis					
Never diagnosed with any	95.49^a^ (0.62)	[0.00; 100.00]	95.56 (0.87)	95.41 (0.88)	*ns*
Diagnosed with any	1.69^b^ (0.20)	[0.00; 69.23]	2.20 (0.28)	1.16 (0.29)	^**^
STI testing frequency					
Perceived recommended frequency	4.89 (0.06)	[1.00; 7.00]	4.99 (0.08)	4.78 (0.08)	*ns*

Different superscripts in each category indicate significant differences with Bonferroni adjustment, all *p* < .001. Percentages may not sum to 100% due to missing responses

^*^p ≤ .050, ^**^p ≤ .010, ^***^p ≤ .001

### Self-Reported STI Knowledge

Results showed overall differences according to STI knowledge, *F*(2, 1480) = 209.42, *p* < .001, η_p_^2^ = .221. Post-hoc comparisons showed that participants had only heard about a higher percentage of STIs and had specific knowledge about a lower percentage of STIs, all *p* < .001 (see Table 2). Even though the difference between regulatory focus groups was non-significant, *F*(2, 740) = 0.75, *p* = .386, η_p_^2^ = .001, there was an interaction between regulatory focus and knowledge, *F*(2, 1480) = 6.79, *p* = .001, η_p_^2^ = .009. Post-hoc comparisons showed that prevention-focused participants had only heard about a higher percentage of STIs, *p* = .007. In contrast, promotion-focused participants indicated they had specific knowledge about a higher percentage of STIs, *p* = .002. No group differences emerged in the percentage of STIs participants had never heard about, *p* = .622.

### Sources of Information about STIs

Results showed no overall differences between sources of information, *F*(2, 994) = 1.40, *p* = .246, η_p_^2^ = .003 (see Table 2), and no differences between regulatory focus groups, *F*(2, 497) = .46, *p* = .830, η_p_^2^ = .000. However, the interaction between regulatory focus and source was significant, *F*(2, 994) = 8.54, *p* < .001, η_p_^2^ = .017. Post-hoc comparisons showed that promotion-focused participants retrieved more of their knowledge from self and peer sources, *p* = .002, and medical sources, *p* = .011, whereas prevention-focused participants retrieved more knowledge from scientific sources, *p* = .033.

### Past STI Testing

Results showed overall differences in past testing, *F*(2, 1480) = 553.59, *p* < .001, η_p_^2^ = .428. Post-hoc comparisons showed that participants had never been tested for a higher percentage of STIs, all *p* < .001, and were unsure of STI testing for a lower percentage of STIs, all *p* < .001 (see Table 2). Again, the difference between regulatory focus groups was non-significant, *F*(2, 740) = 0.12, *p* = .729, η_p_^2^ = .000. However, there was a significant interaction between regulatory focus and past testing, *F*(2, 1480) = 10.02, *p* < .001, η_p_^2^ = .013. Post-hoc comparisons showed that prevention-focused participants had never been tested for a higher percentage of STIs, *p* = .003, whereas promotion-focused participants had been tested for a higher percentage of STIs, *p* < .001. There were no significant differences between the groups in reports of being unsure about past testing, *p* = .762.

### Past STI Diagnosis

Results showed an overall difference in past STI diagnosis, *F*(1, 740) = 17,809.41, *p* < .001, η_p_^2^ = .960, such that participants had never been diagnosed with a higher percentage of STIs (see Table 2). Neither the difference between regulatory focus groups, *F*(1, 740) = 1.00, *p* = .318, η_p_^2^ = .001, nor the interaction between regulatory focus and past diagnosis, *F*(1, 740) = 0.40, *p* = .529, η_p_^2^ = .001,^1^ were significant.

### Recommended Frequency of STI Testing

Results showed no differences between prevention-focused and promotion-focused participants in the perceived recommended frequency with which people should get tested for STIs, *t*(739) = 1.88, *p* = .061, *d* = 0.14.

### Exploratory Analyses

### Sexual Health Practices

Results also showed regulatory focus differences in the reported frequency of past testing and routine sexual health check-ups, both *p* < .001 (see Table 3). Specifically, promotion-focused participants reported they had been tested more often and had more frequent routine sexual health check-ups when compared to prevention-focused participants.

**Table 3:** Differences in sexual health routines and additional contraceptive methods use

	Overall	Regulatory focus group		
		Promotion focus	Prevention focus	Comparisons
	*M* (*SE*) or %	*M* (*SE*) or %	*M* (*SE*) or %	*t* (*d*) or Wald’s *Z*
STI testing				
Frequency of past testing	2.62 (0.07)	3.10 (0.09)	2.14 (0.10)	7.15^***^ (0.53)
Routine sexual health check-ups				
Frequency of past check-ups	2.38 (0.03)	2.53 (0.05)	2.23 (0.05)	4.46^***^ (0.61)
Past STI testing location				
Never tested for STIs	40.6%	31.0%	50.5%	-5.53^***^
Free STI testing facility	15.0%	20.1%	9.6%	4.08^***^
Asked family practice doctor	37.1%	43.4%	30.5%	3.67^***^
Family planning consultation	11.7%	12.4%	11.0%	0.61
Bought a self-testing kit	1.9%	2.1%	1.6%	0.47
Other (e.g., at routine check-ups, before donating blood)	4.4%	4.8%	4.1%	0.42
Other contraceptive methods				
None (other than condoms)	58.5%	53.7%	63.5%	-2.71^**^
Birth control pill	34.9%	39.2%	30.5%	2.49^**^
Intrauterine decide (IUD)	5.9%	6.6%	5.2%	0.81
Other (e.g., vaginal ring)	2.2%	3.2%	1.1%	1.97^*^

^*^p ≤ .050, ^**^p ≤ .010, ^***^p ≤ .001

Moreover, there were group differences in where participants got tested for STIs and which other contraceptive methods they typically use (see Table 3). Promotion-focused participants were more likely to have been tested in free STI testing facilities, *p* < .001, or to ask their family practice doctor to get tested, *p* < .001. They were also more likely to use the birth control pill, *p* = .013, or other contraceptive methods, *p* = .049. In contrast, prevention-focused participants were more likely to have never been tested, *p* < .001, and to use no other contraceptive method, *p* = .007. No other comparisons were significant, *p* ≥ .087.

#### Controlling for Demographics

We explored differences according to our demographic variables of interest and tested if our main analyses were consistent after controlling for these covariates. For the sake of brevity, only statistically significant results are presented, and detailed analyses are available on the Prevent2Protect OSF page.

##### Gender

Results showed that men had never heard about a higher percentage of STIs, *p* = .003, had never been tested for a higher percentage of STIs, *p* < .001, were more likely not to have been tested for STIs in the past, *p* = .005, and to use no other contraceptive method, *p* < .001. Women reported having specific knowledge about a higher percentage of the STIs, *p* = .007, retrieved more of their knowledge from scientific sources, *p* = .028, had been tested for a higher percentage of STIs, *p* < .001, and recommended a higher frequency of STI testing, *p* < .001. Women had also been tested more frequently for STIs, *p* = .001, particularly in family planning consultations, *p* < .001, and using self-testing kits, *p* = .030. Lastly, women had routine check-ups more frequently, *p* < .001, and were more likely to use the birth control pill, *p* < .001. No other differences were significant, *p* ≥ .061.

##### Sexual identity

Results showed that heterosexual participants were more likely to use the birth control pill, *p* = .014. LGBTQI + participants reported having specific knowledge about a higher percentage of STIs, *p* = .021, retrieved more of their knowledge from peer sources, *p* = .024, and recommended a higher frequency of STI testing, *p* = .012. LGBTQI + participants had also been tested more frequently for STIs, *p* = .005, particularly in free testing facilities, *p* = .009, had routine check-ups more frequently, *p* = .008, and were more likely to use no other contraceptive method, *p* = .011. No other differences were significant, *p* ≥ .071.

##### Education

Results showed that less educated participants had never heard about a higher percentage of STIs, *p* < .001, and recommended a higher frequency of STI testing, *p* = .012. More educated people reported having specific knowledge about a higher percentage of the STIs, *p* = .003, and had been tested more frequently, *p* = .041, particularly in free testing facilities, *p* = .007, or other testing locations, *p* = .003. No other differences were significant, *p* ≥ .068.

##### Country

We found that Portuguese participants retrieved more of their knowledge from medical sources, *p* = .005, recommended a higher frequency of STI testing, *p* < .001, and were more likely to have been tested in family planning consultations, *p* = .002, and to use the birth control pill, *p* = .010. Spanish participants were more likely not to have been tested for STIs in the past, *p* = .049, and to use no other contraceptive method, *p* < .001. No other differences were significant, *p* ≥ .061.

##### Controlling for covariates

Significant results from our main analyses (i.e., group differences, interactions, and post-hoc comparisons) remained unchanged after adding these covariates, all *p* ≤ .041. The only exception was that group differences in the likelihood of using other contraceptive methods became non-significant, *p* = .085.

## Discussion

A cross-sectional study with single people living in Portugal and Spain explored if and how people more focused on prevention or promotion differed in their STI knowledge and sexual health practices. Overall, the findings provided mixed support to our hypotheses and revealed interesting nuances.

We found that prevention-focused participants indicated having heard about more STIs, retrieved more of their knowledge from scientific sources, were less likely to have been tested for STIs, and were more likely only to use condoms when having sex. These findings are aligned with past research, considering that people with a prevention focus tend to be warier of health threats and more motivated to seek health information from reliable sources (Rodrigues, 2022; Rodrigues et al., 2019a, 2019b, 2019c; Zou & Scholer, 2016), have more positive attitudes toward condoms use (e.g., perceive condoms as reliable and effective; have pleasure using condoms; Rodrigues & Lopes, 2022), and feel more in control of condom use (Rodrigues et al., 2022). And yet, being motivated toward security and risk aversion in sexual health seems to resonate with being aware or having heard about more STIs, but not necessarily having specific knowledge about more STIs. Although unexpected, this finding may be explained by the sexual health practices enacted by people more focused on prevention. If these people are more likely to use condoms and less likely to get tested for STIs, they may not feel the need to expand their knowledge (e.g., because they feel less susceptible to infections; Rodrigues et al., 2022) and be less exposed to detailed information (e.g., talking less about sexual health with their family practice doctor). From our perspective, perceiving low risk due to condom use and relying on partial STI knowledge can itself create situations that endanger the sexual health of people more focused on prevention. For instance, condom use is a dynamic process, and people may be persuaded to have condomless sex (e.g., Fehr et al., 2015; VanderDrift et al., 2013), and asymptomatic STIs are often left untreated despite being infectious (e.g., Farley et al., 2003).

We also found that promotion-focused participants were more likely to report having specific knowledge about more STIs, retrieve their knowledge from both self and peer as well as medical sources, and have been tested for more STIs. These participants also got tested for STIs more often (either in free testing facilities or after asking their family practice doctor), attended routine sexual health check-ups more often, and were more likely to have used contraceptive methods other than condoms. Research has shown that having a promotion focus is associated with health risk-taking in the pursuit of pleasure (Evans-Paulson et al., 2022; Rodrigues et al., 2022; Zou & Scholer, 2016), particularly when people are more trusting of casual partners (Rodrigues, 2022), and that being worried about becoming infected with STIs and using other contraceptive methods besides condoms increase the odds of STI testing (Thompson et al., 2021). From our perspective, people more focused on promotion seem to engage in a reasoned decision-making process. For example, our findings suggest that these people may negotiate sex without condoms (often perceived as pleasure barriers; Randolph et al., 2007) while maintaining some degree of protection (albeit mostly related to unplanned pregnancies) or at least dealing with potential health consequences afterward (e.g., getting tested for an STI after having sex with someone they perceived as trustworthy). Such a process is inherently risky for the sexual health of people more focused on promotion. Indeed, these people are less likely to use condoms despite being tested for STIs more often and being more exposed to specific knowledge about STIs, either from medical sources (e.g., when they got tested or asked their doctor to get tested) or self and peer sources (e.g., when they search themselves for information or asked others for advice).

The lack of differences in the recommended frequency of STI testing suggests that people focused on either regulatory focus acknowledge the importance of getting tested frequently (or possibly a product of social desirability or norms). The difference, it seems, is that people more focused on prevention may not feel the need to enact subsequent sexual health practices, whereas people more focused on promotion may actively enact such practices, given their condom use patterns.

On a broader note, there were also some demographic differences worth mentioning. We had more women categorized as promotion-focused and more men categorized as prevention-focused. Past evidence has shown that women (vs. men) have less control over condom use and are less comfortable using condoms (Farmer & Meston, 2006; Hall et al., 2019), which is congruent with having a predominant focus on promotion (vs. prevention). However, there is mixed evidence when examining gender differences in risk perception and regulatory focus. For example, some studies suggest that women tend to be more risk averse in health (e.g., Rosen et al., 2003) and knowledgeable about sexual health topics (e.g., Weinstein et al., 2008), but other studies suggest that women are less likely to enact condom negotiation strategies (e.g., Farmer & Meston, 2006; Skakoon-Sparling & Cramer, 2020). We also found that women, LGBTQI + participants, those who received higher education, and Portuguese participants reported having specific knowledge about more STIs, retrieved more of their knowledge from multiple sources, got tested for more STIs, enacted sexual health practices and routines more frequently, and perceived that other people should get tested for STIs more often. In contrast, men, less educated participants, and Spanish participants indicated they had never heard about more STIs and were more likely to have not been tested for more STIs. Some of these findings suggest that people from certain demographic groups (e.g., women, people from sexual minorities, educated people) tend to have better sexual health knowledge (e.g., Grulich et al., 2014; Rodrigues et al., 2020; Weinstein et al., 2008), benefit the most from scientific-based sexual health information (e.g., Fetner et al., 2020; Nikkelen et al., 2020), are more likely to enroll in college sexuality courses (e.g., King et al., 2020), and enact more frequent STI testing (e.g., Thompson et al., 2021). But these findings must be taken with caution. For example, women (vs. men) tend to get tested for STIs more often (and report having more STI knowledge, as we found) but use condoms less often (e.g., Evans-Paulson et al., 2022). Also, people from sexual minorities are more likely to engage in condomless sex (e.g., Kattari et al., 2019; Poteat et al., 2019) and report a higher number of STI diagnoses (e.g., Castro, 2016). However, studies of the intersection of gender and sexual identity show that sexual minority men report more condom use (e.g., Fetner et al., 2020). Lastly, Portuguese and Spanish people may differ in condom use, perceived susceptibility, vulnerability, risk, and self-efficacy (e.g., Muñoz-Silva et al., 2007), but research has shown a consistent negative condom use trend over time (e.g., Ballester-Arnal et al., 2022; Giménez-García et al., 2022; Reis et al., 2018) and a relative lack of STI knowledge in both countries (e.g., Espada et al., 2015; Santos et al., 2018). Notwithstanding, our findings were consistent even after controlling for a priori differences. We believe this speaks to the generalizability of the regulatory focus in sexuality framework across demographics and cultural contexts. Indeed, our study highlights the intricacies of sexual behavior by suggesting that (at least some) demographic differences often reported in the literature are not straightforward and may sometimes be confounded with differences in regulatory focus. Based on our findings, we argue that researchers should consider individual motives and perceptions when examining differences in sexual health decision-making instead of merely relying on demographic characteristics.

### Limitations and Future Research

Our findings should be taken with caution in light of some caveats. As our data were cross-sectional, we were unable to draw inferences regarding causality. Even though we examined regulatory focus in sexuality as a trait-like variable that motivates people to perceive the context and behave in a certain way, research in the health domain has also shown that people can be momentarily induced in a particular regulatory focus (Keller, 2006; Latimer et al., 2008). Hence, future studies could consider regulatory focus malleability and assess if people can shift their predominant focus when facing certain contextual variables (e.g., a negative sexual health experience). Our recruitment efforts returned a large and diverse sample of participants from Portugal and Spain, despite the tendency for a young white urban sample. Future studies could seek to replicate our current finding by following a more diverse (or even representative) sample of participants longitudinally. Such a study would help not only to determine whether regulatory focus influences sexual health decision-making over time, but it could also help determine under which conditions some of the temporal effects are more likely to occur (e.g., trusting the casual partner; Matson et al., 2018; Rodrigues, 2022), or whether people differ their acceptance and use of external and internal condoms (e.g., Kulczycki et al., 2004).

We also asked participants to indicate whether or not they had specific knowledge about, and were tested previously for, each of the 13 STIs. Although our findings could have suffered from social desirability biases, the findings that participants, on average, reported having specific knowledge about only three of the STIs and were unsure of previous testing for only one of the STIs give us confidence in the data herein reported. Still, future research could seek to expand the list of STIs and employ a mixed-methods approach to determine regulatory focus differences in accurate knowledge (including symptomatology, mode of transmission, and course of treatment).

Lastly, our findings also indicate that prevention-focused people were more likely to retrieve their knowledge from scientific sources, including sexual education materials. However, we were unable to determine whether these differences were also a product of the syllabus in sexual education classes. Specifically, most of these classes have a biological approach and emphasize the prevention of risks over sexual pleasure and exploration (e.g., Lameiras-Fernández et al., 2021). Based on our findings, we could argue that prevention-focused people were more attentive and receptive to the typical message conveyed by sexual education (which is aligned with their security motives). In contrast, promotion-focused people may have felt the need to pursue and acquire information elsewhere (e.g., friends with similar pleasure motives). Future studies could explore the extent to which people varying in regulatory focus perceive that distinct sexual education sources (e.g., modules in the syllabus; topics talked about with parents or friends; individual searches in online communities) were important for them and influenced their sexual behavior and decision-making.

### Conclusion

This study added much-needed evidence on the role of individual motives in sexual health decision-making. Extending past research, we found that people more focused on prevention were more aware of more STIs and more likely to have consulted scientific sources, but enacted sexual health behaviors and routines to a lesser extent. In contrast, people more focused on promotion were more knowledgeable of more STIs, more likely to have consulted self and peer and medical sources, and enacted sexual health behaviors more frequently. Our results were largely independent of *a priori* demographic differences between regulatory focus groups, suggesting the generalizability of the framework and the important role of motives for security or pleasure on sexual health decision-making. Hence, these findings are potentially relevant to academics and can inform the revision of theoretical models to predict sexual health more efficiently (or even develop new ones).

More broadly, our findings highlight the potential utility of regulatory focus in sexuality to the development of health messages, campaigns, and interventions to increase sexual health awareness. Sexual health campaigns usually emphasize prevention behaviors (for a review, see Gabarron & Wynn, 2016). However, these campaigns are more effective when the conveyed message is aligned with the person’s regulatory focus (Uskul et al., 2008). As people pursue their health goals and attend to health information differently based on their regulatory focus, campaigns may need to consider having messages that reflect both a prevention focus (e.g., highlight risk awareness and the need to protect oneself and others) *and* a promotion focus (e.g., changing the discourse around condoms to be seen as a pleasurable and fun tool to be used in sex). Given that regulatory focus assessment is a simple process, professionals should also consider making assessments before delivering messages or advice to people in more dynamic approaches (e.g., using mHealth apps). These strategies may help improve behavioral change for a larger number of people by providing them with information adapted to their needs and motives, empowering them to take control over their actions, helping them make more conscious decisions, and improving their overall health and quality of life.

## Supplementary Information

Below is the link to the electronic supplementary material.

Supplementary file1 (DOCX 88 KB)

## Data Availability

All materials, anonymized data, and syntaxes that support our findings are available upon request from the first author and publicly shared on the Prevent2Protect’s OSF page.

## Footnotes

1 Despite the non-significant interaction, post-hoc comparisons showed that promotion-focused participants had been diagnosed with a higher percentage of STIs compared to prevention-focused participants, *p* = .010 (see Table 2).

## References

[CR1] Alvarez-Bruned L, Garcia-Continente X, Gotsens M, Pérez A, Pérez G (2015). Trends in inequalities in the use of condom by urban teenagers in Spain. Journal of Urban Health.

[CR2] Ballester-Arnal R, Gil-Llario MD, Ruiz-Palomino E, Giménez-García C (2017). Effectiveness of a brief multi-component intervention to HIV prevention among Spanish youth. AIDS and Behavior.

[CR3] Ballester-Arnal R, Giménez-García C, Ruiz-Palomino E, Castro-Calvo J, Gil-Llario MD (2022). A trend analysis of condom use in Spanish young people over the two past decades, 1999–2020. AIDS and Behavior.

[CR4] Berinsky AJ, Margolis MF, Sances MW (2014). Separating the shirkers from the workers? Making sure respondents pay attention on self-administered surveys. American Journal of Political Science.

[CR5] Bryan A, Kagee A, Broaddus MR (2006). Condom use among South African adolescents: Developing and testing theoretical models of intentions and behavior. AIDS and Behavior.

[CR6] Byrne B (2012). Structural equation modeling with Mplus: Basic concepts, applications, and programming.

[CR7] Carvalho T, Alvarez M-J, Barz M, Schwarzer R (2015). Preparatory behavior for condom use among heterosexual young men: A longitudinal mediation model. Health Education & Behavior.

[CR8] Carvalho T, Alvarez M-J, Pereira C, Schwarzer R (2016). Stage-based computer-delivered interventions to increase condom use in young men. International Journal of Sexual Health.

[CR9] Castro Á (2016). Sexual behavior and sexual risks among Spanish university students: A descriptive study of gender and sexual orientation. Sexuality Research and Social Policy.

[CR10] Colina S, Marrone N, Ingram M, Sánchez D (2017). Translation quality assessment in health research: A functionalist alternative to back-translation. Evaluation & the Health Professions.

[CR11] Costa ECV, McIntyre T, Trovisqueira A, Hobfoll SE (2017). Comparison of two psycho-educational interventions aimed at preventing HIV and promoting sexual health among Portuguese women. International Journal of Sexual Health.

[CR12] Curran PG (2016). Methods for the detection of carelessly invalid responses in survey data. Journal of Experimental Social Psychology.

[CR13] Espada JP, Morales A, Guillén-Riquelme A, Ballester R, Orgilés M (2016). Predicting condom use in adolescents: A test of three socio-cognitive models using a structural equation modeling approach. BMC Public Health.

[CR14] Espada JP, Morales A, Orgilés M, Jemmott JB, Jemmott LS (2015). Short-term evaluation of a skill-development sexual education program for Spanish adolescents compared with a well-established program. Journal of Adolescent Health.

[CR15] Evans-Paulson R, Widman L, Javidi H, Lipsey N (2022). Is regulatory focus related to condom use, STI/HIV testing, and sexual satisfaction?. Journal of Sex Research.

[CR16] Farley TA, Cohen DA, Elkins W (2003). Asymptomatic sexually transmitted diseases: The case for screening. Preventive Medicine.

[CR17] Farmer MA, Meston CM (2006). Predictors of condom use self-efficacy in an ethnically diverse university sample. Archives of Sexual Behavior.

[CR18] Faul F, Erdfelder E, Buchner A, Lang A-G (2009). Statistical power analyses using G*Power 3.1: Tests for correlation and regression analyses. Behavior Research Methods.

[CR19] Fehr SK, Vidourek RA, King KA (2015). Intra- and inter-personal barriers to condom use among college students: A review of the literature. Sexuality & Culture.

[CR20] Ferrer RA, Lipkus IM, Cerully JL, McBride CM, Shepperd JA, Klein WMP (2017). Developing a scale to assess health regulatory focus. Social Science & Medicine.

[CR21] Fetner T, Dion M, Heath M, Andrejek N, Newell SL, Stick M (2020). Condom use in penile-vaginal intercourse among Canadian adults: Results from the sex in Canada survey. PLoS ONE.

[CR22] Friedman AL, Kachur RE, Noar SM, McFarlane M (2016). Health communication and social marketing campaigns for sexually transmitted disease prevention and control: What is the evidence of their effectiveness?. Sexually Transmitted Diseases.

[CR23] Fuglestad PT, Rothman AJ, Jeffery RW (2013). The effects of regulatory focus on responding to and avoiding slips in a longitudinal study of smoking cessation. Basic and Applied Social Psychology.

[CR24] Gabarron E, Wynn R (2016). Use of social media for sexual health promotion: A scoping review. Global Health Action.

[CR25] Giménez-García C, Ballester-Arnal R, Ruiz-Palomino E, Nebot-García JE, Gil-Llario MD (2022). Trends in HIV sexual prevention: Attitudinal beliefs and behavioral intention in Spanish young people over the past two decades (1999–2020). Sexual & Reproductive Healthcare.

[CR29] Glanz, K., Rimer, B. K., & Viswanath, K. (2015). *Health behavior and health education: Theory, research, and practice* (5th ed.). Jossey-Bass.

[CR26] Grulich AE, de Visser RO, Badcock PB, Smith AMA, Richters J, Rissel C, Simpson JM (2014). Knowledge about and experience of sexually transmissible infections in a representative sample of adults: The Second Australian Study of Health and Relationships. Sexual Health.

[CR27] Guo T, Spina R (2015). Regulatory focus affects predictions of the future. Personality and Social Psychology Bulletin.

[CR28] Hall WJ, Erausquin JT, Nichols TR, Tanner AE, Brown-Jeffy S (2019). Relationship intentions, race, and gender: Student differences in condom use during hookups involving vaginal sex. Journal of American College Health.

[CR30] Higgins ET, Scott RA, Buchmann MC, Kosslyn SM (2015). Regulatory Focus Theory. Emerging Trends in the Social and Behavioral Sciences: An Interdisciplinary, Searchable, and Linkable Resource.

[CR31] Higgins ET, Friedman RS, Harlow RE, Idson LC, Ayduk ON, Taylor A (2001). Achievement orientations from subjective histories of success: Promotion pride versus prevention pride. European Journal of Social Psychology.

[CR32] Kattari SK, Walls NE, Atteberry-Ash B, Klemmer C, Rusow JA, Kattari L (2019). Missing from the conversation: Sexual risk factors across young people by gender identity and sexual orientation. International Journal of Sexual Health.

[CR33] Keller PA (2006). Regulatory focus and efficacy of health messages. Journal of Consumer Research.

[CR34] King BM, Burke SR, Gates TM (2020). Is there a gender difference in US college students’ desire for school-based sexuality education?. Sex Education.

[CR35] Kulczycki A, Kim D-J, Duerr A, Jamieson DJ, Macaluso M (2004). The acceptability of the female and male condom: A randomized crossover trial. Perspectives on Sexual and Reproductive Health.

[CR36] Lameiras-Fernández M, Martínez-Román R, Carrera-Fernández MV, Rodríguez-Castro Y (2021). Sex education in the spotlight: What is working? Systematic review. International Journal of Environmental Research and Public Health.

[CR37] Langens TA (2007). Regulatory focus and illusions of control. Personality and Social Psychology Bulletin.

[CR38] Latimer AE, Rivers SE, Rench TA, Katulak NA, Hicks A, Hodorowski JK, Higgins ET, Salovey P (2008). A field experiment testing the utility of regulatory fit messages for promoting physical activity. Journal of Experimental Social Psychology.

[CR39] Lemarié L, Bellavance F, Chebat J-C (2019). Regulatory focus, time perspective, locus of control and sensation seeking as predictors of risky driving behaviors. Accident Analysis & Prevention.

[CR40] Martin-Smith HA, Okpo EA, Bull ER (2018). Exploring psychosocial predictors of STI testing in university students. BMC Public Health.

[CR41] Matson PA, Fortenberry JD, Chung S, Gaydos CA, Ellen JM (2018). Weekly variations in feelings of trust predict incident STI within a prospective cohort of adolescent women from a US city. Sexually Transmitted Infections.

[CR42] Muñoz-Silva A, Sánchez-García M, Nunes C, Martins A (2007). AIDS prevention in late adolescent college students from Spain and Portugal. Public Health.

[CR43] Muthén, L., & Muthén, B. (2012). *Mplus user’s guide* (Seventh ed.). Author.

[CR44] Nikkelen SWC, van Oosten JMF, van den Borne MMJJ (2020). Sexuality education in the digital era: Intrinsic and extrinsic predictors of online sexual information seeking among youth. Journal of Sex Research.

[CR45] Poteat VP, Russell ST, Dewaele A (2019). Sexual health risk behavior disparities among male and female adolescents using identity and behavior indicators of sexual orientation. Archives of Sexual Behavior.

[CR46] Randolph ME, Pinkerton SD, Bogart LM, Cecil H, Abramson PR (2007). Sexual pleasure and condom use. Archives of Sexual Behavior.

[CR47] Reid AE, Aiken LS (2011). Integration of five health behaviour models: Common strengths and unique contributions to understanding condom use. Psychology & Health.

[CR48] Reis M, Ramiro L, Camacho I, Tomé G, de Matos MG (2018). Trends in Portuguese adolescents’ sexual behavior from 2002 to 2014: HBSC Portuguese study. Portuguese Journal of Public Health.

[CR49] Rodrigues DL (2022). Regulatory focus and perceived safety with casual partners: Implications for perceived risk and casual sex intentions during the COVID-19 pandemic. Psychology & Sexuality.

[CR50] Rodrigues DL, Lopes D (2022). Seeking security or seeking pleasure in sexual behavior? Examining how individual motives shape condom use attitudes.

[CR51] Rodrigues DL, Lopes D, Carvalho AC (2022). Regulatory focus and sexual health: Motives for security and pleasure in sexuality are associated with distinct protective behaviors. Journal of Sex Research.

[CR52] Rodrigues DL, Lopes D, Conley TD (2019). Non-monogamy agreements and safer sex behaviors: The role of perceived sexual self-control. Psychology & Sexuality.

[CR53] Rodrigues DL, Lopes D, Pereira M, Prada M, Garrido MV (2019). Motivations for sexual behavior and intentions to use condoms: Development of the Regulatory Focus in Sexuality scale. Archives of Sexual Behavior.

[CR54] Rodrigues DL, Lopes D, Pereira M, Prada M, Garrido MV (2020). Predictors of condomless sex and sexual health behaviors in a sample of Portuguese single adults. Journal of Sexual Medicine.

[CR55] Rodrigues DL, Prada M, Lopes D (2019). Perceived sexual self-control and condom use with primary and casual sex partners: Age and relationship agreement differences in a Portuguese sample. Psychology & Health.

[CR56] Rosen AB, Tsai JS, Downs SM (2003). Variations in risk attitude across race, gender, and education. Medical Decision Making.

[CR57] Santos MJ, Ferreira E, Duarte J, Ferreira M (2018). Risk factors that influence sexual and reproductive health in Portuguese university students. International Nursing Review.

[CR58] Scott-Sheldon LAJ, Chan PA (2020). Increasing sexually transmitted infections in the U.S: A call for action for research clinical and public health practice [Guest Editorial]. Archives of Sexual Behavior.

[CR59] Skakoon-Sparling S, Cramer KM (2020). Are we blinded by desire? Relationship motivation and sexual risk-taking intentions during condom negotiation. Journal of Sex Research.

[CR60] Soe NMK, Bird Y, Schwandt M, Moraros J (2018). STI health disparities: A systematic review and meta-analysis of the effectiveness of preventive interventions in educational settings. International Journal of Environmental Research and Public Health.

[CR61] Sukhija-Cohen AC, Beymer MR, Engeran-Cordova W, Bolan RK (2019). From control to crisis: The resurgence of sexually transmitted diseases. Sexually Transmitted Diseases.

[CR62] Thompson EL, Griner SB, Galvin AM, Lowery AD, Lewis MA (2021). Correlates of STI testing among US young adults: Opportunities for prevention. Prevention Science.

[CR63] Uskul AK, Keller J, Oyserman D (2008). Regulatory fit and health behavior. Psychology & Health.

[CR64] VanderDrift LE, Agnew CR, Harvey SM, Warren JT (2013). Whose intentions predict? Power over condom use within heterosexual dyads. Health Psychology.

[CR65] Weinstein RB, Walsh JL, Ward LM (2008). Testing a new measure of sexual health knowledge and its connections to students’ sex education, communication, confidence, and condom use. International Journal of Sexual Health.

[CR66] Whiting W, Pharr JR, Buttner MP, Lough NL (2019). Behavioral interventions to increase condom use among college students in the United States: A systematic review. Health Education & Behavior.

[CR67] Wiederman M, Baumeister RF, Vohs KD (2004). Self-control and sexual behavior. Handbook of self- regulation: Research, theory, and applications.

[CR68] Yuan K, Bentler P (2000). Three likelihood-based methods for mean and covariance structure analysis with nonnormal missing data. Sociological Methodology.

[CR69] Zhang W, Wong JYH, Wang T, Fong DYT (2021). University-based behavioral interventions to promote safer sex practices: A systematic review and meta-analysis. Journal of American College Health.

[CR70] Zou X, Scholer AA (2016). Motivational affordance and risk-taking across decision domains. Personality and Social Psychology Bulletin.

