# Supplementary material for: Prevent2Protect Project: Regulatory Focus Differences in Sexual Health Knowledge and Practices
Source: Arch Sex Behav. 2023 Jan 26;52(4):1701–13. doi: 10.1007/s10508-023-02536-3 (PMC9879562; doi:10.1007/s10508-023-02536-3)
Supplement: Supplementary file 1 — Supplementary file1 (DOCX 88 KB) [file 10508_2023_2536_MOESM1_ESM.docx]

**Supplementary Materials**

**S1**

*HIV/AIDS: Results for the main variables (participant level results)*

|  |  | Regulatory focus group | | |
| --- | --- | --- | --- | --- |
|  | Overall | Promotion focused | Prevention focused | Comparisons |
|  | % or *M* (*SE*) | % or *M* (*SE*) | % or *M* (*SE*) | *χ*^2^ (*V*), Wald’s Z, or *t* (*d*) |
| *Self-reported STI knowledge* |  |  |  | 3.58 (0.07) |
| Never heard about | 2.8 | 3.7 | 1.9 |  |
| Only heard about | 37.9 | 35.4 | 40.4 |  |
| Have specific knowledge about | 59.3 | 60.8 | 57.7 |  |
| *Sources of information* |  |  |  |  |
| Self and peer sources | 59.7 | 59.6 | 59.7 | -0.03 |
| Medical sources | 57.7 | 58.9 | 56.4 | 0.57 |
| Scientific sources | 14.8 | 12.1 | 17.8 | -1.80 |
| *Past STI testing* |  |  |  | 11.29^***^ (0.13) |
| Never tested | 58.2 | 52.7^b^ | 63.9^a^ |  |
| Tested | 36.6 | 42.4^a^ | 30.4^b^ |  |
| Unsure of testing | 5.2 | 4.9a | 5.6a |  |
| *Past STI diagnosis* |  |  |  | 0.00 (0.00) |
| Never diagnosed | 99.4 | 99.5 | 99.4 |  |
| Diagnosed | 0.6 | 0.5 | 0.6 |  |
| *STI testing frequency* |  |  |  |  |
| Perceived recommended frequency | 5.57 (0.06) | 5.71^a^ (0.08) | 5.42^b^ (0.09) | 2.59^**^ (0.19) |

*Note.* Sources of information = participants who selected at least one of the sources. Different superscripts (^a^,^b^) indicate significant group differences with Bonferroni adjustment, all *p* ≤ .050. Percentages may not sum to 100% due to missing responses. ^*^*p ≤* .050, ^**^*p* ≤ .010, ^***^*p* ≤ .001

**S2**

*HPV: Results for the main variables*

|  |  | Regulatory focus group | | |
| --- | --- | --- | --- | --- |
|  | Overall | Promotion focused | Prevention focused | Comparisons |
|  | % or *M* (*SE*) | % (*SE*) or *M* (*SE*) | % (*SE*) or *M* (*SE*) | *χ*^2^ (*V*), Wald’s Z, or *t* (*d*) |
| *Self-reported STI knowledge* |  |  |  | 5.07 (0.08) |
| Never heard about | 29.7 | 27.5 | 32.0 |  |
| Only heard about | 44.4 | 43.1 | 45.7 |  |
| Have specific knowledge about | 25.9 | 29.4 | 22.3 |  |
| *Sources of information* |  |  |  |  |
| Self and peer sources | 20.0 | 21.5 | 18.2 | 0.92 |
| Medical sources | 26.3 | 29.8 | 22.5 | 1.87 |
| Scientific sources | 3.4 | 2.3 | 4.7 | -1.48 |
| *Past STI testing* |  |  |  | 14.26^***^ (0.14) |
| Never tested | 68.2 | 62.7^b^ | 74.0^a^ |  |
| Tested | 23.9 | 29.7^a^ | 17.8^b^ |  |
| Unsure of testing | 7.9 | 7.6^a^ | 8.2^a^ |  |
| *Past STI diagnosis* |  |  |  | 2.35 (0.06) |
| Never diagnosed | 95.8 | 94.6 | 97.2 |  |
| Diagnosed | 4.2 | 5.4 | 2.8 |  |
| *STI testing frequency* |  |  |  |  |
| Perceived recommended frequency | 5.00 (0.06) | 5.10 (0.09) | 4.89 (0.09) | 1.69 (0.12) |

*Note.* Sources of information = participants who selected at least one of the sources. Different superscripts (^a^,^b^) indicate significant group differences with Bonferroni adjustment, all *p* ≤ .050. Percentages may not sum to 100% due to missing responses. ^*^*p ≤* .050, ^**^*p* ≤ .010, ^***^*p* ≤ .001

**S3**

*Chlamydia: Results for the main variables*

|  |  | Regulatory focus group | | |
| --- | --- | --- | --- | --- |
|  | Overall | Promotion focused | Prevention focused | Comparisons |
|  | % or *M* (*SE*) | % (*SE*) or *M* (*SE*) | % (*SE*) or *M* (*SE*) | *χ*^2^ (*V*), Wald’s Z, or *t* (*d*) |
| *Self-reported STI knowledge* |  |  |  | 18.80^***^ (0.16) |
| Never heard about | 14.0 | 13.8^a^ | 14.3^a^ |  |
| Only heard about | 57.7 | 51.1^b^ | 64.6^a^ |  |
| Have specific knowledge about | 28.3 | 35.2^a^ | 21.2^b^ |  |
| *Sources of information* |  |  |  |  |
| Self and peer sources | 25.7 | 32.1^a^ | 18.6^b^ | 3.43^***^ |
| Medical sources | 23.6 | 29.1^a^ | 17.4^b^ | 3.08^**^ |
| Scientific sources | 2.8 | 2.3 | 3.4 | -0.76 |
| *Past STI testing* |  |  |  | 15.59^***^ (0.15) |
| Never tested | 70.4 | 64.9^b^ | 76.3^a^ |  |
| Tested | 19.8 | 25.4^a^ | 13.8^b^ |  |
| Unsure of testing | 9.8 | 9.7^a^ | 9.9^a^ |  |
| *Past STI diagnosis* |  |  |  | 8.59^**^ (0.12) |
| Never diagnosed | 95.6 | 93.2^b^ | 98.0^a^ |  |
| Diagnosed | 4.4 | 6.8^a^ | 2.0^b^ |  |
| *STI testing frequency* |  |  |  |  |
| Perceived recommended frequency | 4.95 (0.06) | 5.05 (0.09) | 4.85 (0.09) | 1.64 (0.12) |

*Note.* Sources of information = participants who selected at least one of the sources. Different superscripts (^a^,^b^) indicate significant group differences with Bonferroni adjustment, all *p* ≤ .050. Percentages may not sum to 100% due to missing responses. ^*^*p ≤* .050, ^**^*p* ≤ .010, ^***^*p* ≤ .001

**S4**

*Gonorrhea: Results for the main variables*

|  |  | Regulatory focus group | | |
| --- | --- | --- | --- | --- |
|  | Overall | Promotion focused | Prevention focused | Comparisons |
|  | % or *M* (*SE*) | % (*SE*) or *M* (*SE*) | % (*SE*) or *M* (*SE*) | *χ*^2^ (*V*), Wald’s Z, or *t* (*d*) |
| *Self-reported STI knowledge* |  |  |  | 13.78^***^ (0.14) |
| Never heard about | 6.2 | 7.7^a^ | 4.7^a^ |  |
| Only heard about | 65.3 | 59.0^b^ | 71.9^a^ |  |
| Have specific knowledge about | 28.5 | 33.3^a^ | 23.4^b^ |  |
| *Sources of information* |  |  |  |  |
| Self and peer sources | 25.3 | 30.6^a^ | 19.5^b^ | 2.84^**^ |
| Medical sources | 22.8 | 25.7 | 19.5 | 1.64 |
| Scientific sources | 4.2 | 3.8 | 4.7 | -0.50 |
| *Past STI testing* |  |  |  | 18.16^***^ (0.16) |
| Never tested | 74.8 | 71.1^b^ | 78.6^a^ |  |
| Tested | 16.1 | 21.6^a^ | 10.4^b^ |  |
| Unsure of testing | 9.1 | 7.3^a^ | 11.0^a^ |  |
| *Past STI diagnosis* |  |  |  | 0.48 (0.04) |
| Never diagnosed | 99.0 | 98.6 | 99.4 |  |
| Diagnosed | 1.0 | 1.4 | 0.6 |  |
| *STI testing frequency* |  |  |  |  |
| Perceived recommended frequency | 4.96 (0.06) | 5.07 (0.09) | 4.85 (0.09) | 1.78 (0.13) |

*Note.* Sources of information = participants who selected at least one of the sources. Different superscripts (^a^,^b^) indicate significant group differences with Bonferroni adjustment, all *p* ≤ .050. Percentages may not sum to 100% due to missing responses. ^*^*p ≤* .050, ^**^*p* ≤ .010, ^***^*p* ≤ .001

**S5**

*Hepatitis B: Results for the main variables*

|  |  | Regulatory focus group | | |
| --- | --- | --- | --- | --- |
|  | Overall | Promotion focused | Prevention focused | Comparisons |
|  | % or *M* (*SE*) | % (*SE*) or *M* (*SE*) | % (*SE*) or *M* (*SE*) | *χ*^2^ (*V*), Wald’s Z, or *t* (*d*) |
| *Self-reported STI knowledge* |  |  |  | 7.42^*^ (0.10) |
| Never heard about | 6.1 | 5.3^a^ | 6.9^a^ |  |
| Only heard about | 69.0 | 65.6^b^ | 72.59^a^ |  |
| Have specific knowledge about | 24.9 | 29.1^a^ | 20.6^b^ |  |
| *Sources of information* |  |  |  |  |
| Self and peer sources | 20.8 | 23.4 | 17.8 | 1.54 |
| Medical sources | 21.2 | 24.2 | 17.8 | 1.74 |
| Scientific sources | 3.4 | 2.6 | 4.2 | -0.99 |
| *Past STI testing* |  |  |  | 7.99^*^ (0.11) |
| Never tested | 65.5 | 61.4^b^ | 69.9^a^ |  |
| Tested | 27.3 | 31.9^a^ | 22.5^b^ |  |
| Unsure of testing | 7.2 | 6.8^a^ | 7.6^a^ |  |
| *Past STI diagnosis* |  |  |  | 0.98 (0.05) |
| Never diagnosed | 98.9 | 98.4 | 99.4 |  |
| Diagnosed | 1.1 | 1.6 | 0.6 |  |
| *STI testing frequency* |  |  |  |  |
| Perceived recommended frequency | 4.90 (0.06) | 5.00 (0.09) | 4.78 (0.09) | 1.87 (0.14) |

*Note.* Sources of information = participants who selected at least one of the sources. Different superscripts (^a^,^b^) indicate significant group differences with Bonferroni adjustment, all *p* ≤ .050. Percentages may not sum to 100% due to missing responses. ^*^*p ≤* .050, ^**^*p* ≤ .010, ^***^*p* ≤ .001

**S6**

*Syphilis: Results for the main variables*

|  |  | Regulatory focus group | | |
| --- | --- | --- | --- | --- |
|  | Overall | Promotion focused | Prevention focused | Comparisons |
|  | % or *M* (*SE*) | % (*SE*) or *M* (*SE*) | % (*SE*) or *M* (*SE*) | *χ*^2^ (*V*), Wald’s Z, or *t* (*d*) |
| *Self-reported STI knowledge* |  |  |  | 5.97^*^ (0.09) |
| Never heard about | 4.7 | 5.6^a^ | 3.8^a^ |  |
| Only heard about | 65.8 | 61.6^b^ | 70.1^a^ |  |
| Have specific knowledge about | 29.5 | 32.8^a^ | 26.1^b^ |  |
| *Sources of information* |  |  |  |  |
| Self and peer sources | 27.5 | 31.7^a^ | 22.9^b^ | 2.21^*^ |
| Medical sources | 24.4 | 27.9^a^ | 20.3^b^ | 1.98^*^ |
| Scientific sources | 5.4 | 4.5 | 6.4 | -0.90 |
| *Past STI testing* |  |  |  | 18.99^***^ (0.16) |
| Never tested | 70.0 | 65.6^b^ | 78.6^a^ |  |
| Tested | 19.3 | 26.3^a^ | 13.5^b^ |  |
| Unsure of testing | 7.9 | 8.1^a^ | 7.9^a^ |  |
| *Past STI diagnosis* |  |  |  | 0.77 (0.05) |
| Never diagnosed | 98.6 | 98.1 | 99.1 |  |
| Diagnosed | 1.4 | 1.9 | 0.9 |  |
| *STI testing frequency* |  |  |  |  |
| Perceived recommended frequency | 5.06 (0.06) | 5.20^a^ (0.08) | 4.94^b^ (0.09) | 2.10^*^ (0.15) |

*Note.* Sources of information = participants who selected at least one of the sources. Different superscripts (^a^,^b^) indicate significant group differences with Bonferroni adjustment, all *p* ≤ .050. Percentages may not sum to 100% due to missing responses. ^*^*p ≤* .050, ^**^*p* ≤ .010, ^***^*p* ≤ .001

**S7**

*Genital herpes: Results for the main variables*

|  |  | Regulatory focus group | | |
| --- | --- | --- | --- | --- |
|  | Overall | Promotion focused | Prevention focused | Comparisons |
|  | % or *M* (*SE*) | % (*SE*) or *M* (*SE*) | % (*SE*) or *M* (*SE*) | *χ*^2^ (*V*), Wald’s Z, or *t* (*d*) |
| *Self-reported STI knowledge* |  |  |  | 8.04^*^ (0.10) |
| Never heard about | 4.2 | 3.4^a^ | 4.4^a^ |  |
| Only heard about | 55.7 | 50.5^b^ | 59.8^a^ |  |
| Have specific knowledge about | 39.5 | 46.0^a^ | 35.8^b^ |  |
| *Sources of information* |  |  |  |  |
| Self and peer sources | 36.7 | 39.6 | 33.5 | 1.43 |
| Medical sources | 33.1 | 37.4^a^ | 28.4^b^ | 2.13^*^ |
| Scientific sources | 5.0 | 4.2 | 5.9 | -0.91 |
| *Past STI testing* |  |  |  | 11.62^**^ (0.13) |
| Never tested | 71.6 | 67.3^b^ | 76.1^a^ |  |
| Tested | 19.2 | 24.1^a^ | 14.1^b^ |  |
| Unsure of testing | 9.2 | 8.6^a^ | 9.9^a^ |  |
| *Past STI diagnosis* |  |  |  | 3.09 (0.07) |
| Never diagnosed | 96.0 | 94.6 | 97.4 |  |
| Diagnosed | 4.0 | 5.4 | 2.6 |  |
| *STI testing frequency* |  |  |  |  |
| Perceived recommended frequency | 5.03 (0.06) | 5.10 (0.09) | 4.93 (0.09) | 1.38 (0.10) |

*Note.* Sources of information = participants who selected at least one of the sources. Different superscripts (^a^,^b^) indicate significant group differences with Bonferroni adjustment, all *p* ≤ .050. Percentages may not sum to 100% due to missing responses. ^*^*p ≤* .050, ^**^*p* ≤ .010, ^***^*p* ≤ .001

**S8**

*Trichomoniasis: Results for the main variables*

|  |  | Regulatory focus group | | |
| --- | --- | --- | --- | --- |
|  | Overall | Promotion focused | Prevention focused | Comparisons |
|  | % or *M* (*SE*) | % (*SE*) or *M* (*SE*) | % (*SE*) or *M* (*SE*) | *χ*^2^ (*V*), Wald’s Z, or *t* (*d*) |
| *Self-reported STI knowledge* |  |  |  | 3.34 (0.07) |
| Never heard about | 66.7 | 64.7 | 68.7 |  |
| Only heard about | 26.5 | 26.8 | 26.1 |  |
| Have specific knowledge about | 6.9 | 8.5 | 5.2 |  |
| *Sources of information* |  |  |  |  |
| Self and peer sources | 5.0 | 6.4 | 3.4 | 1.55 |
| Medical sources | 4.4 | 5.3 | 3.4 | 1.03 |
| Scientific sources | 1.0 | 1.1 | 0.8 | 0.32 |
| *Past STI testing* |  |  |  | 9.33^**^ (0.11) |
| Never tested | 76.2 | 71.9^b^ | 80.7^a^ |  |
| Tested | 8.7 | 11.4^a^ | 5.9^b^ |  |
| Unsure of testing | 15.1 | 16.8^a^ | 13.3^a^ |  |
| *Past STI diagnosis* |  |  |  | 0.00 (0.01) |
| Never diagnosed | 98.8 | 98.6 | 98.9 |  |
| Diagnosed | 1.2 | 1.4 | 1.1 |  |
| *STI testing frequency* |  |  |  |  |
| Perceived recommended frequency | 4.57 (0.06) | 4.63 (0.09) | 4.51 (0.09) | 0.97 (0.08) |

*Note.* Sources of information = participants who selected at least one of the sources. Different superscripts (^a^,^b^) indicate significant group differences with Bonferroni adjustment, all *p* ≤ .050. Percentages may not sum to 100% due to missing responses. ^*^*p ≤* .050, ^**^*p* ≤ .010, ^***^*p* ≤ .001

**S9**

*Hepatitis A: Results for the main variables*

|  |  | Regulatory focus group | | |
| --- | --- | --- | --- | --- |
|  | Overall | Promotion focused | Prevention focused | Comparisons |
|  | % or *M* (*SE*) | % (*SE*) or *M* (*SE*) | % (*SE*) or *M* (*SE*) | *χ*^2^ (*V*), Wald’s Z, or *t* (*d*) |
| *Self-reported STI knowledge* |  |  |  | 5.81 (0.09) |
| Never heard about | 8.5 | 8.2 | 8.8 |  |
| Only heard about | 69.9 | 66.7 | 73.4 |  |
| Have specific knowledge about | 21.6 | 25.1 | 17.9 |  |
| *Sources of information* |  |  |  |  |
| Self and peer sources | 17.8 | 19.2 | 16.1 | 0.92 |
| Medical sources | 16.4 | 20.0^a^ | 12.3^b^ | 2.33^*^ |
| Scientific sources | 3.4 | 3.0 | 3.4 | -0.49 |
| *Past STI testing* |  |  |  | 12.21^**^ (0.13) |
| Never tested | 67.5 | 62.1^b^ | 73.2^a^ |  |
| Tested | 23.5 | 28.7^a^ | 18.0^b^ |  |
| Unsure of testing | 9.0 | 9.2^a^ | 8.7^a^ |  |
| *Past STI diagnosis* |  |  |  | 0.23 (0.03) |
| Never diagnosed | 98.2 | 97.8 | 98.6 |  |
| Diagnosed | 1.8 | 2.2 | 1.4 |  |
| *STI testing frequency* |  |  |  |  |
| Perceived recommended frequency | 4.82 (0.06) | 4.95^a^ (0.09) | 4.69^b^ (0.09) | 2.10^*^ (0.16) |

*Note.* Sources of information = participants who selected at least one of the sources. Different superscripts (^a^,^b^) indicate significant group differences with Bonferroni adjustment, all *p* ≤ .050. Percentages may not sum to 100% due to missing responses. ^*^*p ≤* .050, ^**^*p* ≤ .010, ^***^*p* ≤ .001

**S10**

*Hepatitis C: Results for the main variables*

|  |  | Regulatory focus group | | |
| --- | --- | --- | --- | --- |
|  | Overall | Promotion focused | Prevention focused | Comparisons |
|  | % or *M* (*SE*) | % (*SE*) or *M* (*SE*) | % (*SE*) or *M* (*SE*) | *χ*^2^ (*V*), Wald’s Z, or *t* (*d*) |
| *Self-reported STI knowledge* |  |  |  | 5.43 (0.09) |
| Never heard about | 5.1 | 5.3 | 4.9 |  |
| Only heard about | 68.3 | 64.6 | 72.3 |  |
| Have specific knowledge about | 26.5 | 30.2 | 22.8 |  |
| *Sources of information* |  |  |  |  |
| Self and peer sources | 22.4 | 23.8 | 20.8 | 0.81 |
| Medical sources | 19.2 | 21.9 | 16.1 | 1.64 |
| Scientific sources | 3.4 | 2.6 | 4.2 | -0.99 |
| *Past STI testing* |  |  |  | 11.54^**^ (0.13) |
| Never tested | 65.4 | 60.3^b^ | 70.7^a^ |  |
| Tested | 26.5 | 31.9^a^ | 20.8^b^ |  |
| Unsure of testing | 8.1 | 7.8^a^ | 8.5^a^ |  |
| *Past STI diagnosis* |  |  |  | 0.00 (0.02) |
| Never diagnosed | 99.6 | 99.5 | 99.7 |  |
| Diagnosed | 0.4 | 0.5 | 0.3 |  |
| *STI testing frequency* |  |  |  |  |
| Perceived recommended frequency | 4.92 (0.06) | 5.01 (0.09) | 4.78 (0.09) | 1.90 (0.14) |

*Note.* Sources of information = participants who selected at least one of the sources. Sources of information = participants who selected at least one of the sources. Different superscripts (^a^,^b^) indicate significant group differences with Bonferroni adjustment, all *p* ≤ .050. Percentages may not sum to 100% due to missing responses. ^*^*p ≤* .050, ^**^*p* ≤ .010, ^***^*p* ≤ .001

**S11**

*Cytomegalovirus: Results for the main variables*

|  |  | Regulatory focus group | | |
| --- | --- | --- | --- | --- |
|  | Overall | Promotion focused | Prevention focused | Comparisons |
|  | % or *M* (*SE*) | % (*SE*) or *M* (*SE*) | % (*SE*) or *M* (*SE*) | *χ*^2^ (*V*), Wald’s Z, or *t* (*d*) |
| *Self-reported STI knowledge* |  |  |  | 0.42 (0.02) |
| Never heard about | 69.5 | 68.4 | 70.6 |  |
| Only heard about | 23.9 | 24.7 | 23.1 |  |
| Have specific knowledge about | 6.6 | 6.9 | 6.3 |  |
| *Sources of information* |  |  |  |  |
| Self and peer sources | 4.8 | 4.9 | 4.7 | 0.13 |
| Medical sources | 4.0 | 3.8 | 4.2 | -0.27 |
| Scientific sources | 1.6 | 1.5 | 1.7 | -0.17 |
| *Past STI testing* |  |  |  | 5.02 (0.08) |
| Never tested | 77.2 | 74.1 | 80.6 |  |
| Tested | 8.4 | 10.3 | 6.5 |  |
| Unsure of testing | 14.3 | 15.7 | 13.0 |  |
| *Past STI diagnosis* |  |  |  | 0.00 (0.02) |
| Never diagnosed | 99.0 | 99.2 | 98.9 |  |
| Diagnosed | 1.0 | 0.8 | 1.1 |  |
| *STI testing frequency* |  |  |  |  |
| Perceived recommended frequency | 4.60 (0.06) | 4.67 (0.09) | 4.47 (0.09) | 1.60 (0.12) |

*Note.* Sources of information = participants who selected at least one of the sources. Different superscripts (^a^,^b^) indicate significant group differences with Bonferroni adjustment, all *p* ≤ .050. Percentages may not sum to 100% due to missing responses. ^*^*p ≤* .050, ^**^*p* ≤ .010, ^***^*p* ≤ .001

**S12**

*Mycoplasma genitalium: Results for the main variables*

|  |  | Regulatory focus group | | |
| --- | --- | --- | --- | --- |
|  | Overall | Promotion focused | Prevention focused | Comparisons |
|  | % or *M* (*SE*) | % (*SE*) or *M* (*SE*) | % (*SE*) or *M* (*SE*) | *χ*^2^ (*V*), Wald’s Z, or *t* (*d*) |
| *Self-reported STI knowledge* |  |  |  | 0.44 (0.02) |
| Never heard about | 72.4 | 71.7 | 73.1 |  |
| Only heard about | 21.6 | 21.7 | 21.4 |  |
| Have specific knowledge about | 6.1 | 6.6 | 5.5 |  |
| *Sources of information* |  |  |  |  |
| Self and peer sources | 4.6 | 4.2 | 5.1 | -0.50 |
| Medical sources | 4.6 | 4.9 | 4.2 | 0.36 |
| Scientific sources | 1.4 | 1.1 | 1.7 | -0.54 |
| *Past STI testing* |  |  |  | 4.31 (0.08) |
| Never tested | 77.9 | 74.8 | 81.2 |  |
| Tested | 7.0 | 8.1 | 5.9 |  |
| Unsure of testing | 15.0 | 17.1 | 12.9 |  |
| *Past STI diagnosis* |  |  |  | 0.00 (0.00) |
| Never diagnosed | 98.9 | 98.9 | 98.9 |  |
| Diagnosed | 1.1 | 1.1 | 1.1 |  |
| *STI testing frequency* |  |  |  |  |
| Perceived recommended frequency | 4.61 (0.06) | 4.65 (0.09) | 4.52 (0.09) | 1.03 (0.08) |

*Note.* Sources of information = participants who selected at least one of the sources. Different superscripts (^a^,^b^) indicate significant group differences with Bonferroni adjustment, all *p* ≤ .050. Percentages may not sum to 100% due to missing responses. ^*^*p ≤* .050, ^**^*p* ≤ .010, ^***^*p* ≤ .001

**S13**

*Lymphogranuloma venereum: Results for the main variables*

|  |  | Regulatory focus group | | |
| --- | --- | --- | --- | --- |
|  | Overall | Promotion focused | Prevention focused | Comparisons |
|  | % or *M* (*SE*) | % (*SE*) or *M* (*SE*) | % (*SE*) or *M* (*SE*) | *χ*^2^ (*V*), Wald’s Z, or *t* (*d*) |
| *Self-reported STI knowledge* |  |  |  | 0.03 (0.01) |
| Never heard about | 74.1 | 74.3 | 73.8 |  |
| Only heard about | 21.1 | 20.9 | 21.2 |  |
| Have specific knowledge about | 4.9 | 4.8 | 5.0 |  |
| *Sources of information* |  |  |  |  |
| Self and peer sources | 4.0 | 3.8 | 4.2 | -0.27 |
| Medical sources | 3.6 | 3.4 | 3.8 | -0.25 |
| Scientific sources | 0.8 | 0.4 | 1.3 | -1.12 |
| *Past STI testing* |  |  |  | 2.15 (0.05) |
| Never tested | 77.6 | 75.4 | 79.9 |  |
| Tested | 6.6 | 7.3 | 5.9 |  |
| Unsure of testing | 15.7 | 17.3 | 14.1 |  |
| *Past STI diagnosis* |  |  |  | 0.00 (0.02) |
| Never diagnosed | 99.6 | 99.7 | 99.4 |  |
| Diagnosed | 0.4 | 0.3 | 0.6 |  |
| *STI testing frequency* |  |  |  |  |
| Perceived recommended frequency | 4.60 (0.06) | 4.65 (0.09) | 4.53 (0.09) | 0.95 (0.07) |

*Note.* Sources of information = participants who selected at least one of the sources. Different superscripts (^a^,^b^) indicate significant group differences with Bonferroni adjustment, all *p* ≤ .050. Percentages may not sum to 100% due to missing responses. ^*^*p ≤* .050, ^**^*p* ≤ .010, ^***^*p* ≤ .001
